# Compliance with D2 lymph node dissection in reduced-port totally laparoscopic distal gastrectomy in patients with gastric cancer

**DOI:** 10.1038/s41598-021-83386-8

**Published:** 2021-02-11

**Authors:** Sung Eun Oh, Jeong Eun Seo, Ji Yeong An, Min-Gew Choi, Tae Sung Sohn, Jae Moon Bae, Sung Kim, Jun Ho Lee

**Affiliations:** 1 Department of Surgery, Samsung Medical Center, Sungkyunkwan University School of Medicine, 81 Irwon-ro, Gangnam-gu, Seoul, 06351 Korea; 2 Present Address: Department of Surgery, Hallym University Sacred Heart Hospital, Anyang-si, Gyeonggi-do Korea; 3 Present Address: Department of Surgery, Changwon Samsung Medical Center, Changwon-si, Gyeongsangnam-do Korea

**Keywords:** Gastrointestinal cancer, Surgical oncology

## Abstract

This phase II clinical trial was performed to determine whether reduced-port laparoscopic surgery with complete D2 lymph node (LN) dissection for gastric cancer is a safe and feasible surgical technique. The prospectively enrolled 65 gastric cancer patients underwent reduced-port surgery (i.e., triple-incision totally laparoscopic distal gastrectomy [Duet TLDG] with D2 lymphadenectomy). Compliance rate was the primary outcome, which was defined as cases in which there was no more than one missing LN station during D2 LN dissection. The secondary outcomes were the numbers of dissected and retrieved LNs in each station and other short-term surgical outcomes and postoperative course. The compliance rate was 58.5%. The total number of retrieved LNs was 41 (range: 14–83 LNs). The most common station missing from LN retrieval was station no. 5 (35/65; 53.8%), followed by station no. 1 (24/65; 36.9%). The overall postoperative complication rate was 20.0% (13/65). One patient underwent surgical treatment for postoperative complications. There was no instances of mortality. Duet TLDG is an oncologically and technically safe surgical method of gastrectomy and D2 lymphadenectomy.

## Introduction

Laparoscopic surgery for the treatment of early gastric cancer was proven to be an oncologically and technically safe procedure. The five-year survival rate of patients who received laparoscopic gastrectomy did not significantly differ from those of open gastrectomy, while the complication rate was significantly lower for laparoscopic gastrectomy^1,2^.Therefore, according to Korean practice guidelines, laparoscopic gastrectomy with D1 + lymph node (LN) dissection is the first option for the treatment of early gastric cancer^3^.

However, in cases of advanced gastric cancer, pursuing a laparoscopic approach remains controversial due to the need for meticulous LN dissection. Given D2 LN dissection is the standard operating approach for advanced gastric cancer in Korea and Japan^3,4^, there has been some effort to apply minimally invasive techniques in the treatment of advanced gastric cancer. Clinical trials tested the oncological feasibility of laparoscopic gastrectomy with D2 LN dissection^5,6^, finding that compliance rates (i.e., cases when there was no more than one missing LN station during D2 LN dissection) were similar between open and laparoscopic surgeries. In COACT 1001 study, which was a randomized phase II multicenter clinical trial, evaluated 196 patients (100 in laparoscopy group and 96 in open group) and concluded that there were no significant differences in the overall noncompliance rate of LN dissection between those two groups (laparoscopy 47.0% and open 43.2%; P = 0.648)^6^.

Previous studies have found that reduced-port laparoscopic surgeries were not inferior in terms of surgical outcome relative to conventional laparoscopic surgeries for gastric cancer^7,8^. We also recently developed the technique of reduced-port (three-port) totally laparoscopic distal gastrectomy (Duet TLDG) with the goal of limiting patient wound pain and procedural costs^9^. Because the median number of dissected LNs and the number of the dissected LNs in each LN station did not differ between Duet TLDG and conventional laparoscopic-assisted distal gastrectomy, this surgical procedure was feasible in terms of patient safety and the quality of D1 + LN dissection for early gastric cancer^10^.

Before expanding Duet TLDG to treat patients with advanced gastric cancer, the possibility of achieving complete D2 lymphadenectomy should be investigated in clinical studies. Thus, the purpose of this phase II clinical trial was to determine whether Duet TLDG with complete D2 LN dissection for gastric cancer is an oncologically feasible treatment approach.

## Materials and methods

### Patients

We prospectively enrolled 68 patients with gastric cancer between December 2014 and November 2019. Patients who were planned to undergo Duet TLDG with D2 LN dissection at Samsung Medical Center with a histological diagnosis of adenocarcinoma of the stomach; a Eastern Cooperative Oncology Group performance status of 0 or 1; age of between 20 and 80 years; location of the primary tumor in the antrum, angle, or low body; and clinical stage T1-3NxM0, which was determined with preoperative abdominal-pelvic computed tomography, endoscopy, and chest X-ray, were eligible for inclusion in this study.

We excluded patients with metastasis, double primary cancer, those receiving palliative treatment, and those who underwent the resection of another organ during gastrectomy. Ultimately, three patients were excluded from the analysis: one had peritoneal seeding at the diaphragm, another was converted to open surgery due to severe adhesion caused by a previous operation, and the third underwent total gastrectomy to achieve an adequate proximal resection margin. All enrolled patients submitted written informed consent prior to entering the study. We performed the present study according to the Declaration of Helsinki and the study protocol was approved by the institutional review board of Samsung Medical Center, Seoul, Korea (SMC 2014–10-032). Clinical trial registration information: URL for Clinical Trial https://cris.nih.go.kr, Date of registration 16/07/2020, Registration Number for Clinical Trial KCT0005241.

### Surgical procedures

**Figure 1:**
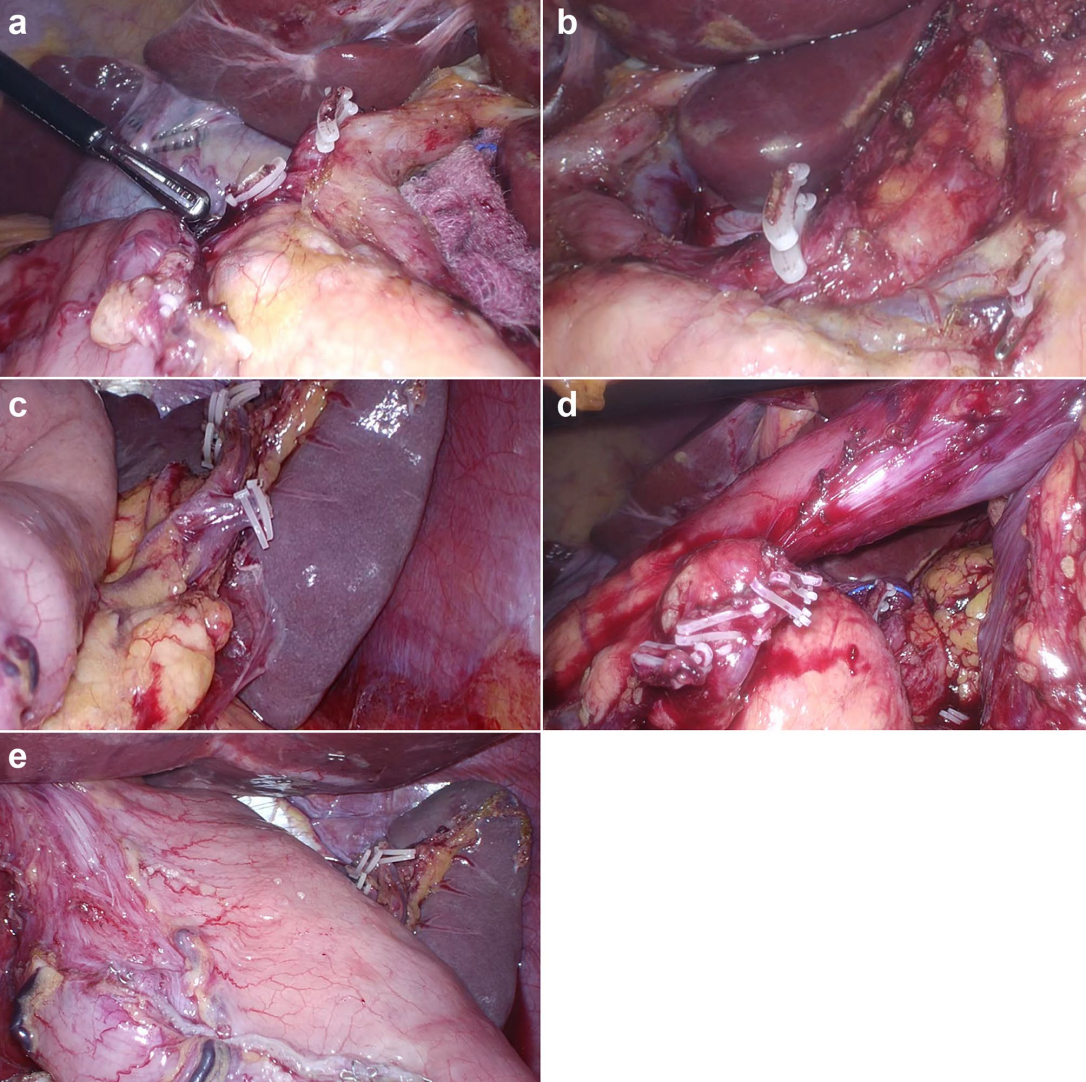
The surgical field of D2 lymph node dissection during reduced-port totally laparoscopic gastrectomy. **(a)** Ligated right gastric artery and supraduodenal artery (station no. 5). **(b)** Ligated left gastric artery and posterior gastric artery (station no. 7, 8a, 9, 11p). **(c)** Ligated left gastroepiploic artery (station no. 4sb). **(d)** Ligated right gastroepiploic artery (station no. 6). **(e)** Dissection of station no. 1, 3.

All Duet TLDGs were performed by a single surgeon and a laparoscopist^9^. Two 12-mm trocars were inserted at the umbilicus and left subcostal area, respectively: one was used as a working trocar (umbilicus) and the other was used for the insertion of the laparoscope (left subcostal). Additionally, a 5-mm trocar was inserted at the right lower flank for instruments held in the surgeon’s left hand. Following retraction of the liver with 1–0 nylon suture, total omentectomy and D2 LN dissection (Fig. 1) were conducted. Then, after radical distal gastrectomy and specimen retrieval, anastomosis was performed in intracorporeal Billroth II gastrojejunostomy with linear staplers.

### Histopathological examination

After the operation, the operator examined the resected specimens macroscopically. The retrieved LNs were immediately categorized into LN stations as defined by the Japanese classification of gastric carcinoma^11^ on the back table by a medical laboratory technologist (Ahn GD). All specimens were sent to the department of pathology after the operation and all pathological examinations were completed in a routine manner. Pathologic stage was classified according to the eighth edition of the American Joint Committee on Cancer classification^12^.

### Primary and secondary outcomes

The primary study outcome was the compliance rate, defined as cases in which there was no more than one missing LN station during D2 LN dissection (D1 + No. 8a,9,11p,12a)^4,13^, while the secondary outcomes included the numbers of dissected LNs in each station, cases in which less than 25 LNs were retrieved, operation time, estimated blood loss (mL), postoperative complications (recorded according to the Clavien–Dindo classification), and postoperative in-hospital days.

### Sample size

We predicted a compliance rate of 67.2% would be achieved in this study similar to as was reported in a previous study examining conventional laparoscopic distal gastrectomy with D2 LN dissection^5^. The sample size was based on the alpha error at 0.05 and a power of 90%. As we set the equivalence difference to be 20% between the conventional laparoscopic distal gastrectomy and Duet TLDG, the total sample size required was calculated to be 60 patients according to the formula^14^. When we added 10% to mitigate expected follow-up loss, the total sample size was calculated to be 67 patients.

### Statistical analysis

An equivalence test (two one-sided tests)^15^ of a one-sample proportion was performed to compare the compliance rate of this study and the rate of a previous study^5^. We confirmed that the compliance rate of this study showed no significant difference by more than 20% from the previous result if the p-value was less than 0.05. Statistical analysis was carried out using the statistical software SAS version 9.4 (SAS Institute, Cary, NC, USA) and SPSS version 22.0 for Windows (IBM Corp., Armonk, NY, USA).

## Results

Table 1 presents clinicopathologic characteristics of the patients and tumors. More male patients (56.9%) than female patients (43.1%) were included. The median age was 56 years (range: 21–76 years) and the median body mass index was 22.9 kg/m^2^ (range: 16.3–29.2 kg/m^2^). Cancers were mostly located in the antrum (49.2%) and low body (29.2%). Among the 65 study participants, 58 (89.2%) underwent Billroth II anastomosis. Pathological examination showed that 49.2% of cases involved advanced gastric cancers. The proportions of stage II and stage III disease were 26.2% and 13.8%, respectively.

**Table 1:** Characteristics of the patients and tumors.

Characteristics	Patients	
	Number	%
**Sex**		
Male	37	56.9
Female	28	43.1
**Age (years)**		
Median	56	
Range	21–76	
**Body mass index (kg/m**^**2**^)		
Median	22.9	
Range	16.3–29.2	
**Anastomosis**		
Billroth I	7	10.8
Billroth II	58	89.2
**Tumor size (mm)**		
Mean	38	
Range	10–100	
**Tumor location**		
Antrum	32	49.2
Angle	4	6.2
Low body	19	29.2
Midbody	10	15.4
**Lauren classification**		
Intestinal	20	30.8
Diffuse	33	50.8
Mixed	11	16.9
Indeterminate	1	1.5
**Depth of invasion**		
T1	33	50.8
T2	14	21.5
T3	11	16.9
T4	7	10.8
**LN metastasis**		
N0	47	72.3
N1	8	12.3
N2	5	7.7
N3	5	7.7
**Stage**		
I	39	60.0
II	17	26.2
III	9	13.8

Short-term surgical outcomes and postoperative course are shown in Table 2. The operating time was 162 min (range: 118–228 min) and the estimated blood loss that occurred during surgery was 100 mL (range: 30–450 mL). The length of hospital stay was nine days (range: 7–19 days), while 80.0% of patients showed no postoperative complications and 18.5% of patients experienced Clavien–Dindo grades I or II complications. Only one patient underwent reoperation due to anastomosis leakage, which was at the common entry hole, the insertion site of linear stapler arm. There was a 3 mm perforation in the anastomosis site. After the primary repair of the anastomosis site, antibiotics were treated, and upper gastrointestinal series were performed before the start of diet. The patient was discharged after 15 days without further complication. There was no case of mortality in this study.

**Table 2:** Short-term surgical outcomes and postoperative course.

Outcomes	Patients	
	Number	%
**Retrieved LNs (number)**		
Median	41	
Range	14–83	
**Compliance of LN retrieval**		
Complete	38	58.5
Incomplete	27	41.5
**Operation time (minutes)**		
Median	162	
Range	118–228	
**Blood loss during operation (ml)**		
Median	100	
Range	30–450	
**Hospital stay (days)**		
Median	9	
Range	7–19	
**Postoperative complication (CD classification)**		
None	52	80.0
I	7	10.8
II	5	7.7
III	1	1.5

The total number of retrieved LNs was 41 (range: 14–83 LNs) (Table 2). The compliance rate was 58.5% (*p* = 0.025). We rejected the null hypothesis at a significance level of 0.05. In other words, the rejection of the null hypothesis means that the calculated p is included within the reference value of 0.672 and margin. There was no difference by more than 20% from the compliance rate (67.2%) of the previous study^5^.

**Figure 2:**
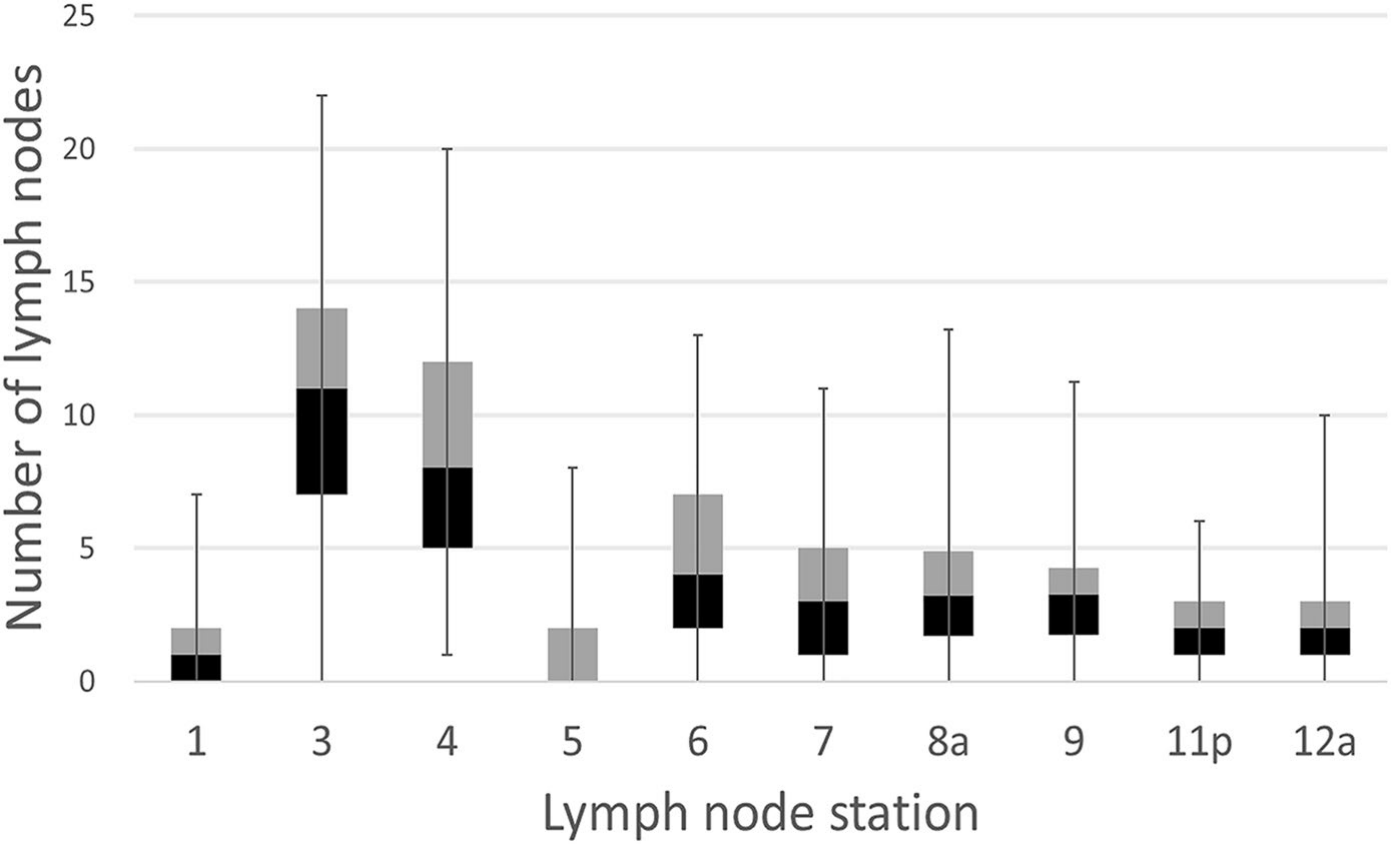
The number of dissected lymph nodes at each lymph node station. The most common station missing from lymph node retrieval at pathological examination was station no. 5 (35/65, 53.8%), followed by station no. 1 (24/65, 36.9%). However, station no. 4 was retrieved in all 65 cases.

Less than 25 LNs were retrieved in five patients (5/65; 7.7%), including one patient in whom fewer than 16 LNs were retrieved. The most common station missing from LN retrieval at the time of pathological examination was station no. 5 (35/65; 53.8%), followed by station no. 1 (24/65; 36.9%). However, station no. 4 was retrieved in all 65 cases (Fig. 2).

## Discussion

The compliance rate of D2 LN dissection through Duet TLDG, which was the primary outcome of this prospective study, was 58.5%. The median number of total retrieved LNs was 41 and more than 25 LNs were retrieved in 92.3% of 65 patients.

D2 LN dissection is a part of the standard surgical armamentarium for the treatment of patients with advanced gastric cancer^3,4^. The proportion of patients with advanced gastric cancer (T2 or more) in this study was 49.2%, which was higher than that in other previous clinical trials involving D2 LN dissection with conventional laparoscopic gastrectomy^5^. To confirm the completeness of D2 LN dissection with Duet TLDG, the compliance rate was used for comparison, based on that of another previous study^13^. As there might be biological variations in the number of LNs in each station^16^, we allowed for one missing station per case.

Compliance rates of conventional laparoscopic gastrectomy reported by previous studies include 67.2%^5^ and 53%^6^.This study was performed as a phase II equivalence trial^17^ and aimed to show that conventional laparoscopic gastrectomy and Duet TLDG were not too different in the conduct of D2 lymphadenectomy (67.2% vs. 58.5%). The compliance rate of this study was not significantly different by more than 20% in comparison with the previously reported compliance rate (67.2%) of conventional laparoscopic surgery. Although there was no statistically significant difference between the two compliance rates, the discrepancy in these results might reflect a difference in the performance of the surgeon, pathologist, and institution involved.

To evaluate the completeness of D2 lymphadenectomy, we also examined the total number of retrieved LNs. In a previous study, the retrieval of more than 25 nodes in advanced, node-negative gastric cancer patients was suggested to be the right number of nodes retrieved by an “adequate” D2 lymph node dissection^18^. In this regard, we confirmed that more than 25 nodes were retrieved in all of the compliant patients in this study. The total number of dissected LNs in each patient alongside the compliance rate may demonstrate the level of technical completion in D2 LN dissection of this study.

The most common station missing from LN retrieval was station no. 5 and station no. 1. However, station no. 4 was retrieved in all cases. The right paracardia nodes (station no. 1) are located on the right side of the cardia, along the first ramification of the ascending branch of the left gastric artery (cardia-esophageal branch). Suprapyloric nodes (station no. 5) are located at the lesser curvature, immediately above the pylorus, along the right gastric artery and its origin. Lastly, the greater curvature nodes (station no. 4) are located along the left and right gastroepiploic arteries. Although there might be developed lymphatic plexus, the number of LNs in station no. 1 and 5 may be few or none than other station due to less anatomical space than other LN stations. In addition, there might be technically difficulty in complete dissection of station no. 1 and 5 during Duet TLTG because the direction of laparoscopic view is from the left side of the patient and to the right. On the other hand, as we performed total omentectomy, the LNs in station no. 4 might be fully dissected.

The postoperative complication rate was 20.0%; however, most cases were treated with conservative treatment. A previous study suggested that the degree of LN dissection (D1 + versus D2) is an important risk factor of complications during and after laparoscopic gastrectomy^19^. In this study, we experienced one case of open conversion due to severe adhesion after open abdominal surgery but not due to major vessel injury during LN dissection and no instances of postoperative vascular complications, which might be a concern for surgeons during LN dissection. Although, from our results, Duet TLDG seems to be a safe procedure, one caveat to keep in mind is that the operator gained experience in Duet TLDG while performing D1 + dissection before conducting this study. It is still important for surgeons to remain cautious when performing D2 dissection in Duet TLDG.

Conventional laparoscopic gastrectomy has become a standard method for patients with early gastric cancer due to the improved quality of life that results relative to following open surgery and its comparable long-term survival outcomes^20^. As the laparoscopic technique has evolved, the number of ports used for laparoscopic gastrectomy has been reduced to one to three and the operator no longer needs to involve an assistant during their surgeries^7–9,21,22^. Reducing the number of ports may lower postoperative wound pain^23^, limit port-related complications, and have a positive effect on the cosmetic aspect after surgery. Furthermore, it may lessen the necessary manpower and surgery-related costs. The median duration of hospital stay in this study was nine days. During this period, we prepared for surgery for 2 days after admission. Therefore, most patients who underwent Duet TLDG were discharged a week after surgery. The median postoperative hospital stay of Duet TLDG was similar with conventional laparoscopic group (7 [7–23] days versus 7 [6–9], *p* = 0.423)^10^. In other study, there was no significant difference between the conventional laparoscopic group and reduced port surgery group regarding length of hospital stay (8.8 days versus 7.9, *p* = 0.233)^24^. Although surgeons can reduce hospitalization for patients who have undergone conventional laparoscopic or reduced port surgery than open gastrectomy, research should be performed to develop an effective clinical pathway for reduced port surgery to reduce hospitalization than conventional laparoscopic surgery.

The quality of oncological aspects, especially LN dissection, in reducing port surgery (Duet TLDG) for early gastric cancer was previously confirmed^10^. As D2 LN dissection is known to be possible in cases of conventional laparoscopic distal gastrectomy^5,25^, we conducted this study to confirm the possibility of D2 lymphadenectomy in Duet TLDG.

There are limitations in this study. Although this is phase II clinical trial, the sample size is quite low and there is no comparative group. Large sample size is required and should be accompanied by comparative study with conventional laparoscopy group for more confirmative conclusion about this surgical technique. As all Duet TLDG procedures were performed by one surgeon in a single center, the applicability of this technique among other surgeons, institutions, and patient populations should be further investigated. Also, there might have been contamination that occurred during categorizing the dissected LNs into the station. In this study, station no. 1 and 5 may be categorized to no. 3, which result in the station no. 1 and 5 the most common station missing from LN retrieval. For further accurate categorization, a marking procedure during the operation is necessary.

In conclusion, Duet TLDG is shown to be feasible and safe in this study. However, a randomized prospective clinical trial to compare survival and other surgical outcomes would be necessary to validate this technique in the treatment of patients with advanced gastric cancer.
